# Sex and weaponry: The distribution of toxin‐storage glands on the bodies of male and female cane toads (*Rhinella marina*)

**DOI:** 10.1002/ece3.2914

**Published:** 2017-09-22

**Authors:** Wei Chen, Cameron M. Hudson, Jayna L. DeVore, Richard Shine

**Affiliations:** ^1^ School of Life and Environmental Sciences University of Sydney Sydney NSW 2006 Australia; ^2^ Ecological Security and Protection Key Laboratory of Sichuan Province Mianyang Normal University Mianyang 621000 China

**Keywords:** antipredator, bufadienolide, *Bufo marinus*, bufotoxin, chemical defense, sexual dimorphism

## Abstract

The parotoid macroglands of bufonid anurans store (and can expel) large volumes of toxic secretions and have attracted detailed research. However, toxins also are stored in smaller glands that are distributed on the limbs and dorsal surface of the body. Female and male cane toads (*Rhinella marina*) differ in the location of toxin‐storage glands and the extent of glandular structures. Female toads store a larger proportion of their toxins in the parotoids than males as well as (to a lesser extent) in smaller glands on the forelimbs. Males have smaller and more elongate parotoids than females, but glands cover more of the skin surface on their limbs (especially hindlimbs) and dorsal surface. The delay to toxin exudation in response to electrostimulation varied among glands in various parts of the body, and did so differently in males than in females. The spatial distribution of toxin glands differs between the sexes even in toads that have been raised under standardized conditions in captivity; hence, the sexual dimorphism is due to heritable factors rather than developmentally plastic responses to ecological (e.g., habitat, predation risk) differences between the sexes. The selective advantages of this sexual dimorphism remain unclear. A priori, we might expect to see toxin widely dispersed across any part of the body likely to be contacted by a predator; and a wide distribution also would be expected if the gland secretions have other (e.g., male–male rivalry) functions. Why, then, is toxin concentrated in the parotoids, especially in female toads? That concentration may enhance the effectiveness of frontal displays to deter predation and also may facilitate the transfer of stored toxins to eggs.

## 1 Introduction

Although they share many genes in common, males and females within a population often differ in a wide range of phenotypic traits. The most easily explained divergences involve characteristics with a direct role in reproductive biology, such as gonads, accessory glands, and sex‐specific ornaments or weaponry (e.g., antlers in male deer: Clutton‐Brock, 1991; Andersson, 1994). However, the sexes also differ in a wide range of traits with no obvious link to reproduction. For example, males and females sometimes forage on different kinds of prey or feed in different places using different tactics; and such ecological divergence can result in the evolution of sexual dimorphism in size or shape of the trophic apparatus (Fairbairn, Blanckenhorn, & Székely, 2007; Neuhaus & Ruckstuhl, 2005; Shine, 1989). Even the sensory modalities used to detect prey (e.g., Vincent, Shine, & Brown, 2005) and cognitive abilities of the sexes (Carazo, Noble, Chandrasoma, & Whiting, 2014) are subject to differential selective pressures.

Morphological divergence between the sexes is widespread in anuran amphibians, and often involves attributes such as mean adult body sizes and relative limb lengths (reflecting sexual selection and fecundity selection: Shine, 1979; Lee, 2001; Kupfer, 2007; Wells, 2010). Direct physical combat between males has favored the evolution of sex‐specific weaponry such as pseudo‐fangs and spines (Katsikaros & Shine, 1997; Emerson, 2001; Tsuji & Matsui, 2002; Hudson, He, & Fu, 2010) and increased musculature of the forelegs (Navas & James, 2007). Although antipredator tactics generally are similar between male and female anurans within the same population, species (and even populations) differ considerably in traits such as the possession of defensive chemicals (Brodie, Ridenhour, & Brodie, 2002), and color polymorphism in many tropical anurans may have evolved in antipredator contexts (see Wells, 2010 for a review). Nonetheless, we are unaware of any examples whereby conspecific male and female amphibians differ strongly in morphological traits that function to deter predation. The closest example may be the shift away from crypsis toward bright coloration by male anurans at the peak of breeding activity (Doucet & Mennill, 2009).

Given that general lack of sexual dimorphism in antipredator tactics, we were surprised to notice a difference between male and female cane toads (*Rhinella marina* Linnaeus 1758) in the distribution of toxin‐secreting glands across the body. First, casual observation suggests that glands are more numerous in the dorsal skin of adult males than females. Second, field and laboratory observations indicate that stressed male toads sometimes exude toxin over most of their dorsal surface, whereas females mostly exude toxin from the parotoid glands. Lastly, when we attempted to extract toxin from the parotoids of toads (to use as an attractant in tadpole trapping: Crossland, Haramura, Salim, Capon, & Shine, 2012), we obtained more exudate from the parotoids of females than of males. Those perplexing observations encouraged us to quantify the distribution of toxin‐containing glands in male and female toads.

Histological studies have documented the morphology of bufonid skin glands in great detail (e.g., Hostetler & Cannon, 1974; Jared et al., 2009; Schwinger, Zanger, & Greven, 2001; Toledo & Jared, 1995). Most research has focused on the parotoid macroglands (which consist of around 120–130 secretary units: Hutchinson & Savitzky, 2004), to the neglect of smaller glands in other parts of the body. Nonetheless, the existence of these additional glands is well known. For example, Regueira, Dávila, and Hermida (2016, p. 14–15) noted that “granular glands from the big warts in the skin of *R. [Rhinella] arenarum* produce toxins with similar characteristics to that of parotoid glands, that is, catecholamines and lipid‐derived secretions, but do not display the same organization as the macroglands.” The same authors indicated that “we did not observe differences between males and females for the studied skin regions [in the trunk region].” The ventral skin of bufonids contains mucous‐producing but not toxin‐producing glands (Regueira et al., 2016), so was not included in our study.

## 2 Materials and Methods

### 2.1 Electrostimulation trials

This component of the study was conducted on 13 adult toads, all from northeastern New South Wales (Brooms Head; Table 1). We conducted electrostimulation trials on 16 standardized locations across the dorsal surface of each toad (to check that glands in the skin of the dorsum and limbs of both male and female toads exude toxin if stimulated and measure the delay prior to secretion). Those locations were bilaterally symmetrical, with eight sites on each side of the body (dorsal surface of upper and lower forelimbs; dorsal surface of upper and lower hindlimbs; three evenly spaced sites on either side of the dorsal midline; and parotoid macrogland).

**Table 1:** Collection locations and specimens examined

Site	Latitude	Longitude	*n*	Female		*n*	Male	
				Mean (mm)	Range (mm)		Mean (mm)	Range (mm)
Wild‐caught toads								
Durack	15°56′43.74″S	127°13′16.81″E	9	110.8	92–128	10	105.2	87–117
Ellenbrae	15°58′27.64″S	127°03′43.70″E	12	116.8	106–134	5	116.2	110–120
Innisfail	17°31′28.85″S	146°01′56.38″E	10	113.3	105–120	12	110.3	100–121
Richmond	20°43′46.04″S	143°08′30.10″E	10	130.8	118–142	13	117.1	102–125
Katherine	14°26′20.39″S	132°16′18.67″E	14	130.3	115–144	11	121.4	102–132
Oombulgurri	15°10′49.50″S	127°50′42.14″E	14	119.2	111–136	11	112.0	107–117
Tully	17°55′58.33″S	145°55′24.80″E	11	112.9	94–147	10	98.9	83–114
Townsville	19°15′27.44″S	146°49′4.36″E	13	116.0	105–127	10	104.2	96–113
Mt. Isa	20°43′28.94″S	139°29′50.86″E	10	105.1	87–123	15	103.8	95–115
Jabiru	12°40′15.54″S	132°50′23.33″E	11	130.2	120–135	14	119.7	110–135
Brooms Head	29°36′29.65″S	153°20′08.83″E	6	109.0	100–123	7	103.5	93–120
Captive‐raised progeny								
El Questro	16°00′84.38″S	127°97′98.11″E	4	105.3	61–120	1	100	100
Innisfail	17°31′28.85″S	146°01′56.38″E	16	131.2	53–219	15	110	54–184
Oombulgurri	15°10′49.50″S	127°50′42.14″E	11	98.7	56–158	6	96.0	53–170
Purnululu	17°52′97.52″S	128°40′08.38″E	3	76.7	37–99	3	78.5	73–83
Townsville	19°15′27.44″S	146°49′4.36″E	13	116.9	58–203	16	96.4	52–138
Tully	17°55′58.33″S	145°55′24.80″E	14	123.1	69–194	23	94.2	50–175
Wyndham	15°46′48.03″S	128°10′01.43″E	3	90.9	61–129	6	104.8	75–152

Data for wild‐caught adult toads (including specimens from Richmond that were used for electrostimulation trials as well as morphological measurements) and also for captive‐raised progeny of wild‐caught toads.

We used a purpose‐built electrostimulator to deliver toxin (Lindley, 1969) using a maximum stimulation time of 240 s with 10 V and 130 Hz, delivered via platinum electrodes separated by 10 mm. We did not wet the skin prior to testing, and the probe was held immobile throughout the trial. During the trial period, we recorded the time prior to the first appearance of toxin (milky fluid) on the skin surface. If no toxin appeared within 240 s, we scored the response as “did not exude.”

### 2.2 Gland attributes

For each toad used in the electrostimulation trials, we also scored the following traits:

1. Surface area and shape of the parotoid macroglands—We photographed all toads and then used the image‐analysis freeware ImageJ (Rasband, W.S., ImageJ, U.S. National Institutes of Health, Bethesda, MD, USA) to calculate surface area of the macroglands. From the same photographs, we recorded maximum length and width (mm) of each parotoid macrogland.
2. Proportion of the skin surface covered by glandular structures—We calculated the proportion of glandular structures for each toad by pressing a transparent microscope slide marked with a 10 × 10 mm quadrat onto the toad's body and scoring the proportion of that quadrat that was glandular. These measurements were taken at the same locations where electrostimulus trials were conducted. To confirm the presence of toxin‐producing granular glands in the skin, we also took histological samples from the dorsal tissues of a small subset of toads (four males and two females).

### 2.3 Field‐caught animals

We also took the standardized measurements above on 238 adult cane toads (120 females, 118 males; 83–147 mm snout‐urostyle length; SUL), collected from 11 locations spanning the eastern to western edges of the species’ invasive distribution in Australia (Table 1). We included these geographically diverse samples to minimize impacts of any local variation and to ensure that our results apply broadly. All patterns were consistent across populations, although the magnitude of sexual dimorphism sometimes differed. We do not describe these geographic effects in the present study, because (together with sexual dimorphism in other traits) they are the subject of a separate study (C. M. Hudson, W. Chen, & R. Shine, in prep.).

### 2.4 Captive‐raised progeny

We repeated these morphological measurements on 134 adult (78–125 mm SUL) cane toads that had been raised in our field station from the egg stage (see Table 1 for details). Field‐caught adults had been spawned at the field station using hormonal priming, and the resultant offspring maintained under standard conditions (see Hudson, Phillips, Brown, & Shine, 2015 for more information on husbandry methods).

### 2.5 Statistical analyses

Using JMP Pro version 11.2.0 (SAS Institute, Cary, NC), we conducted an ANOVA with toad sex and area of body (forelimb, hindlimb, dorsum, parotoid) as the factors, plus their interaction, and the delay to exude toxin (ln‐transformed to attain normality and variance homogeneity) as the dependent variable, and with individual ID # as a random factor to account for repeated measures on the same animals. Because this analysis identified a significant interaction term, we proceeded to look at data for males and females separately, using ANOVA with location on the body as the factor, and toad ID as a random factor. For analyses of morphological traits, we used an ANCOVA with sex as the factor, toad body size (SUL) as the covariate, and the morphological measure (value per region per toad) as the dependent variable.

## 3 Results

### 3.1 Electrostimulation trials

**Figure 1:**
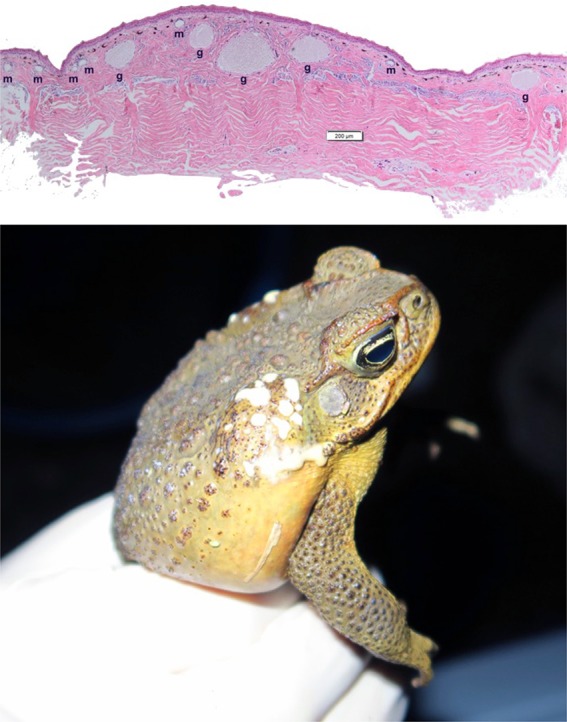
Histological sample of male dorsal skin depicting both granular (g) and mixed (m) glands, and a male cane toad secreting toxin from its parotoid macroglands. Photographs by G. Brown and C. Shilton (upper panel) and J. DeVore and C. Hudson (lower panel)

All of the body parts we identified as “glandular” exuded milky fluid when stimulated, suggesting that these are all toxin‐exuding glands (Figure 1).

**Figure 2:**
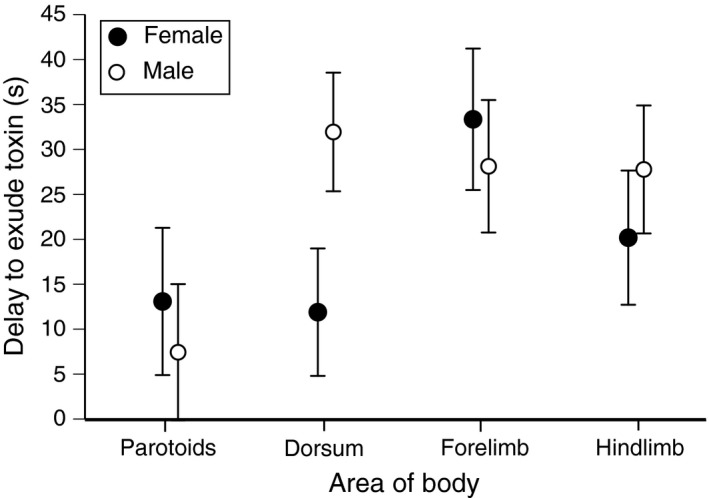
Delay prior to secretion of milky fluid from skin glands following standardized electrostimulation of various parts of the body of male and female cane toads (*Rhinella marina*). The graph shows mean values and associated standard errors for toxin release times for glands on the toad's dorsal surface and forelimbs and hindlimbs as well as the parotoid macroglands

The time taken from the onset of electrostimulation to milky fluids being exuded ranged from 3 to 110 s (mean ± 1 SEM: 20.73 ± 1.75 s, *n *=* *181 readings). The time taken prior to exudation differed among areas of the body (forelimbs, hindlimbs, dorsum, parotoids) in different ways in males versus females (interaction sex*area of body, *F*_3,163.4_ = 7.25, *p *<* *.0001). That significant interaction term reflects a trend for parotoid glands to exude more rapidly in males than in females, whereas the reverse was true for dorsal glands (Figure 2). If we analyze the data separately for each sex, the mean time to exude toxin was higher for limb glands than for dorsal or parotoid glands in females (*F*_3,79.11_ = 3.05, *p* < .04; Tukey's post hoc tests show delay is significantly longer for forelimbs than for dorsal glands). In males, mean time to exude toxin was lowest for parotoids (*F*_3,85.71_ = 11.73, *p* < .001; Tukey's post hoc shows that delay is significantly lower for parotoids than for all other glands).

### 3.2 Gland attributes of field‐caught animals

Male and female toads differed in the relative size of the parotoid macroglands, and in the proportion of skin surface covered by toxin‐secreting glands in other parts of the body. These patterns in sexual dimorphism were evident within all populations that we examined.

**Figure 3:**
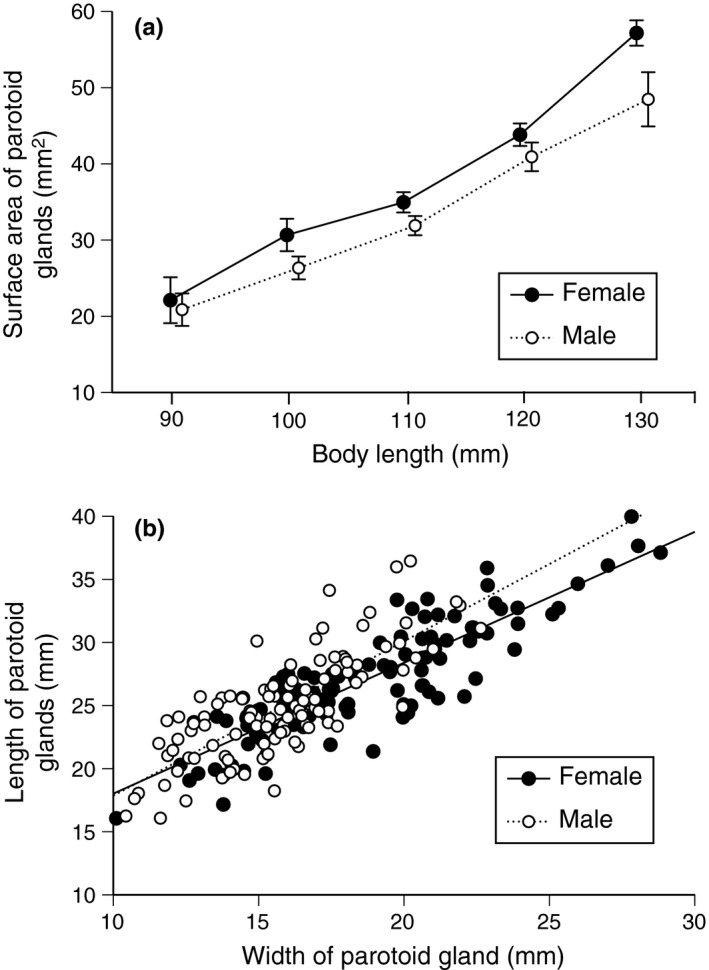
The size (surface area) and shape of parotoid glands of male and female cane toads as a function of body length. Panels show (a) surface area of parotoid macroglands relative to toad body length, and (b) divergence between the sexes in the shape (maximum length relative to maximum width) of parotoid macroglands in wild‐caught cane toads, *Rhinella marina*. The graph shows mean values and associated standard errors for gland surface area for each 10‐mm size class in snout‐urostyle length. The extreme values (90 and 130 mm) include a few individuals slightly smaller (for 90 mm) or larger (for 130 mm) than the stated sizes

**Figure 4:**
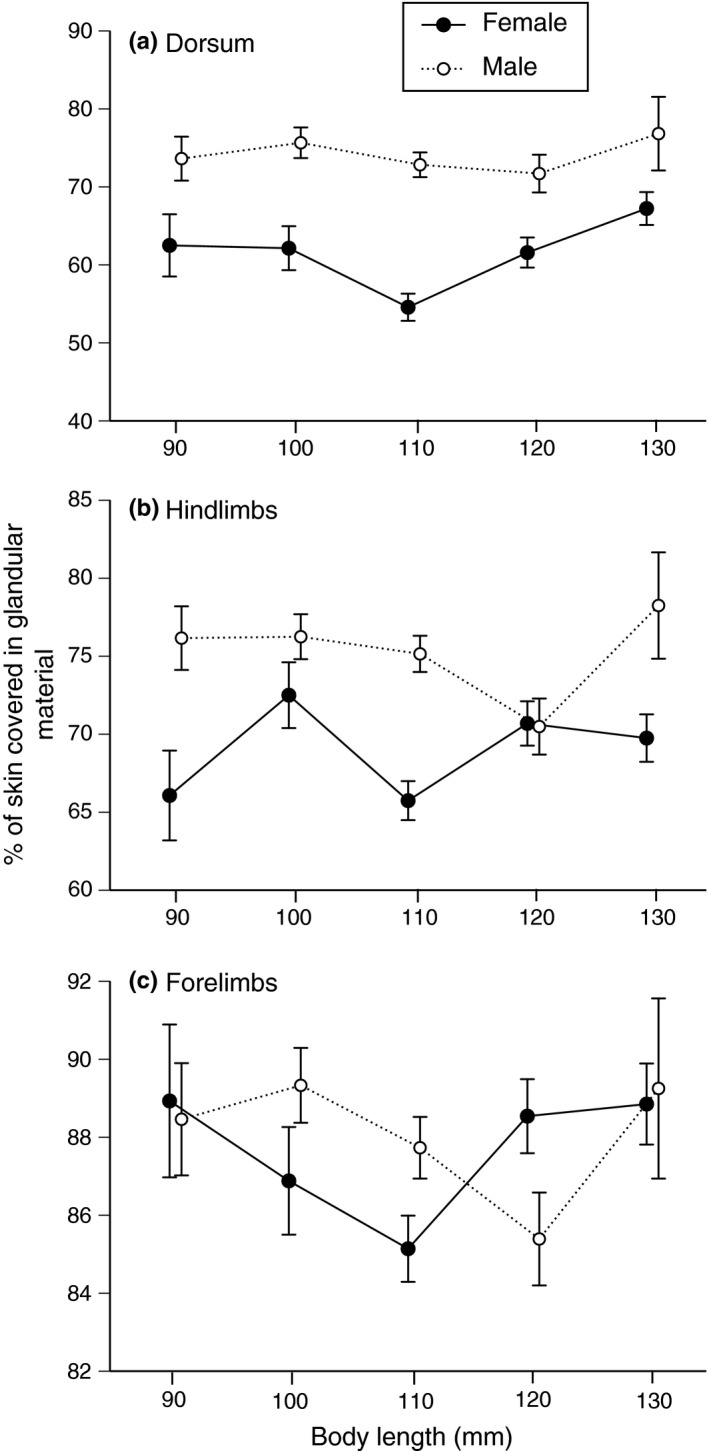
Differences between male and female cane toads in the proportion of the skin covered in glandular structures in sections of the (a) dorsum, (b) hindlimbs, and (c) forelimbs

On average, males had smaller parotoid macroglands than did females both in absolute terms, and relative to body size (interaction sex*SUL, *F*_1,210_ = 4.52, *p *<* *.04; main effect of sex, *F*_1,210_ = 7.08, *p *<* *.01; see Figure 3a). Additionally, the parotoid glands of male toads were more elongate than were those of females (ANCOVA with sex as the factor, macrogland width [mean of left and right glands] as the covariate, mean macrogland length as the dependent variable; interaction sex*SUL, *F*_1,214_ = 2.95, *p *=* *.09; sex effect, *F*_1,214_ = 10.98, *p *<* *.002; Figure 3b). However, the proportion of the body surface covered by glandular structures was higher in males than females in other areas of the body. The sexual disparity was significant for the dorsum (*F*_1,215_ = 76.57, *p *<* *.0001; Figure 4a) and hindlimbs (*F*_1,215_ = 28.84, *p *<* *.0001; Figure 4b) but not the forelimbs (*F*_1,218_ = 0.56, *p *=* *.45; Figure 4c). Histological samples confirmed that both granular and mixed glands were present in the dorsal skin of both sexes (Figure 1).

### 3.3 Gland attributes of captive‐raised animals

Our measurements of captive‐raised progeny showed patterns almost identical to those in wild‐caught animals (above). Specifically, males had smaller parotoid macroglands than did females (interaction sex*SUL, NS in all cases; main effect of sex, *F*_1,130_ = 8.33, *p *<* *.005), whereas the proportion of the body surface covered by glandular structures was higher in males than females for the dorsum (*F*_1,132_ = 135.24, *p *<* *.0001), the hindlimbs (*F*_1,132_ = 24.78, *p *<* *.0001), and the forelimbs (*F*_1,132_ = 15.58, *p *<* *.0001).

As in field‐caught animals, males had more elongate parotoids than did females (ANCOVA with sex as the factor, macrogland width as the covariate, macrogland length as the dependent variable; interaction sex*SUL, *F*_1,131_ = 1.46, *p *=* *.23; sex effect, *F*_1,131_ = 5.05, *p *<* *.03).

## 4 Discussion

Despite an extensive (mostly, histologically‐based) literature on the skin glands of bufonids, the existence of strong sexual dimorphism in the size and location of toxin‐containing glands appears to have escaped notice. Our data show that male cane toads have smaller and more elongate parotoid macroglands than females of the same body size, but greater glandular coverage on the dorsum and limbs. All of these glands secrete milky fluid under electrostimulation, suggesting that all are indeed reservoirs of toxin. Future work could usefully examine the chemical composition of the fluid released by different regions in the body in male and female toads; although it appeared similar (and was reported to be approximately the same chemically by Regueira et al., 2016), more detailed analysis would be of interest. The amount of electrical stimulation required to elicit exudate differed among glands and also differed (in some cases) between male and female toads stimulated at the same location on the body.

What mechanisms are responsible for these sexual differences? One possibility is that these divergences are nonadaptive consequences of the profound physiological (e.g., endocrine) differences between male and female toads. Particular parts of the body might respond differently to such factors. Alternatively, the morphological differences between the sexes might be driven by developmental plasticity. Experimental manipulations have revealed that many aspects of anuran morphology are highly plastic (Kearney, Pell, Byrne, & Reina, 2014; Relyea, 2001). Directly relevant to the current study, Hagman, Hayes, Capon, and Shine (2009) reported that the size of the parotoid macroglands in metamorph cane toads is increased by exposure to alarm cues during larval life. Although male and female tadpoles presumably encounter similar environments, sensitivity to developmental conditions later in life might generate sexual differences in gland sizes. Adult male and female cane toads utilize available habitats differently; for example, females are often found in densely vegetated sites far from water, whereas males congregate around breeding ponds (González‐Bernal, Brown, Crowther, & Shine, 2015). Abiotic or biotic differences between their resultant experiences (e.g., in operative temperatures or in exposure to predation) might directly modify morphological development, including investment into the parotoids and other glands. However, the magnitude and consistency of male–female differences, and the persistence of those differences even in captive‐raised offspring (with no opportunity to select sex‐specific habitats) argue strongly against this interpretation.

Lastly (and most consistent with our data), sexual dimorphism in the distribution of toxin‐containing glands may have evolved because of adaptation: that is, the sexes have evolved different spatial distributions of these glands across their bodies because of the way that gland size and distribution affects an individual toad's viability. The factors generating that sexual disparity in fitness consequences remain unclear, but we suggest the following possibilities:

1. Differences between the sexes in vulnerability to predation—Adult male toads that call from exposed sites near water bodies may be vulnerable to avian predators (Beckmann & Shine, 2011) and hence benefit from a wide distribution of toxin across the dorsal surface (the part of the body most likely to be contacted by an aerial predator). In contrast, females that live in thicker vegetation may be more at risk from terrestrial predators such as rodents (González‐Bernal et al., 2015) and snakes (Shine, 2010), that are best repelled by a frontal display that exposes the parotoids and forelimb glands prominently.
2. The need for females to redeploy toxin to developing eggs—The eggs of cane toads contain high levels of several bufadienolides (Hayes, Crossland, Hagman, Capon, & Shine, 2009). If these chemicals are synthesized in the parotoids from cholesterol precursors (Hutchinson & Savitzky, 2004), they may then need to be transferred through the bloodstream to the ovaries. Concentrating the site of toxin storage (especially in a site like the parotoids, relatively close to the heart and supplied by major blood vessels) might facilitate such a redeployment.
3. Male‐male rivalry—Male toads compete vigorously for access to females, with rival males often usurping an already‐amplexed animal; those wrestling bouts likely confer strong selection on a male's ability to cling to his partner (and thus explain the seasonal swelling of forearm musculature and nuptial spines in male anurans: Shine, 1979; Wells, 2010). The dorsal skin of male *R. marina* becomes spinose during the breeding season, and may assist males to deter other male from displacing them during amplexus. Because the initially amplectant male clings firmly to the female, a newly‐arriving male ends up on top of the first male – a position that causes the spinose skin of the first male to push directly against the thin ventral skin (including pelvic patch) of the potential usurper. Exudates from glands in that area could further repel the rival; the relatively slow release of toxin from male dorsal glands fits well with this interpretation. Information on the chemical composition of these exudates (irritants rather than toxins?) would be of great interest.
4. Osmotic or hydric balance—The contents of these glands include not only bufadienolides, but also a diverse array of biogenic amines and glycosaminoglycans (Clarke, 1997). All of these substances may play wider biological roles in osmotic balance and/or hydric balance (Dapson, 1970; Elkan, 1968; Le Quang Trong, 1975a,b; Lichtstein, Gati, Haver, & Katz, 1992; Lillywhite, 1971; Matoltsy & Bereiter‐Hahn, 1986; Toledo & Jared, 1995; Toledo, Jared, & Brunner, 1992; Vialli, Bolognani, Croce, & Bolognani, 1969). Thus, the toxin‐containing glands may serve additional functions other than deterrence of predators. In some anurans (but apparently not in cane toads: Schwinger et al., 2001), males and females differ in skin thickness (Greven, Zanger, & Schwinger, 1995, for *Xenopus laevis*); such a difference might impose selection on a range of functions that are conferred by glandular secretions and on the structures that produce glandular secretions.

A priori, we might expect an animal that deters predators by chemical means to deploy that defensive tissue widely across the body. Such a distribution would maximize the probability of a predator encountering the repellent regardless of where on the toad's body it directs its initial attack. In that sense, the concentration of toxins within the parotoid macroglands of bufonids is surprising. However, at least some predators are likely to be detected by the toad before they launch an attack, allowing the toad to orient itself to face the oncoming predator and inflate its lungs (both increasing its apparent size and [by providing firm pressure from beneath] facilitating emptying of toxin‐storage glands under pressure from the predator: Mailho‐Fontana et al., 2014). If toads can detect approaching predators in enough time for the anuran to orient toward the threat, a concentration of toxin in the anterior part of the body may maximize the arsenal exposed to the predator's initial onslaught.

More broadly, interspecific as well as intraspecific (sex‐based) divergences in the amount and location of toxin‐storage glands warrant additional research. Within the “true toads” (Bufonidae), for example, morphologically similar species vary enormously in the relative size of the parotoid glands, and in whether or not toxin‐secreting glands also occur on the limbs (Blair, 1972; Bücherl & Buckley, 1971). To our knowledge, that variation has never been examined in any comprehensive framework. Given the strong allometry of parotoid size in cane toads (Phillips & Shine, 2006a), and divergences in relative parotoid size among populations of cane toads within Australia (Phillips & Shine, 2006b), it would be of great interest to explore relationships between a species’ body size, ecology, and its investment into toxin production and storage. Differences between the sexes in size, ecology, and toxin gland location equally would be worth investigating, as would intraspecific (geographic) variation in such traits. For example, do bufonids with smaller parotoids have more toxins distributed around their bodies, in smaller glands (as is the case with male versus female cane toads)?

For a comprehensive understanding of variation in investment into chemical defenses, we also would need to quantify not only the store of toxin within a toad's body, but also the rate at which that store can be replenished after it is used against a predator (e.g., see Jared et al., 2014 for this approach). Plausibly, a sex or species or life‐history stage capable of replenishing toxin supplies more rapidly could “afford” to maintain a lower store of toxin. Future research also could explore factors that affect the toad's willingness to exude its toxin stores under natural (or simulated) conditions. In our experience, cane toads generally rely on crypsis or escape if approached by a predator, and rarely express toxin unless they are under severe duress. However, individuals with severe spinal arthritis (and hence less capable of sustained locomotion) soon cease attempting to move away from the threat and instead resort to defensive displays with toxin secretion (Brown, Shilton, Phillips, & Shine, 2007). It would be relatively straightforward to investigate the effects of a toad's sex, size, location, and previous experience, as well as weather conditions, in determining the animal's propensity to deploy toxins at the skin surface when harassed. In short, we know a great deal about the detailed morphology of toxin‐storage glands in anurans, but far less about the ways in which the animals actually use those toxins for defense against predators. The strong sexual dimorphism in location of toxin stores in cane toads is intriguing and hints that ecological factors may influence the tactics used by anurans to store and deploy their formidable chemical weaponry.

## Conflict of Interest

None declared.

## Data Accessibility

Data will be deposited in the Dryad repository.
